# Long-term survival after surgical resection for bone metastasis from pancreatic cancer: A case report

**DOI:** 10.1097/MD.0000000000035856

**Published:** 2023-11-17

**Authors:** Koki Hayashi, Minoru Kitago, Yuta Abe, Hiroshi Yagi, Yasushi Hasegawa, Shutaro Hori, Masayuki Tanaka, Yutaka Nakano, Keisuke Asakura, Yohei Masugi, Yuko Kitagawa

**Affiliations:** a Department of Surgery, Keio University School of Medicine, Shinjuku, Japan; b Department of Thoracic Surgery, Keio University School of Medicine, Shinjuku, Japan; c Department of Pathology, Keio University School of Medicine, Shinjukusss, Japan.

**Keywords:** bone metastasis, conversion surgery, pancreatic cancer

## Abstract

**Introduction:**

Pancreatic cancer (PC) is highly malignant and metastatic; however, bone metastases are rare. Although the effectiveness of conversion surgery for distant metastases of PC has been reported in a few cases, there are no reports on surgical resection for bone metastases. Here, we report a case of long-term survival after resection of bone metastasis from PC.

**Patient concerns:**

A 60-year-old woman underwent pancreaticoduodenectomy after neoadjuvant chemoradiotherapy for pancreatic head cancer. At 28 months after surgery, multiple lung metastases from PC were diagnosed, and chemotherapy was administered. After 59 months, chemotherapy was terminated because all target lesions had disappeared on imaging.

**Diagnosis:**

At 77 months after the initial surgery, bone metastasis in the left 9^th^ rib was detected by positron emission tomography/computed tomography, which was performed due to elevated carbohydrate antigen 19-9 levels.

**Interventions:**

Chemotherapy was readministered as the initial treatment. Subsequently, due to the long-term well-controlled status of the recurrence site and the absence of other metastases, thoracoscopic-assisted partial resection of the left 9^th^ rib was performed 128 months following pancreaticoduodenectomy. Pathological examination revealed adenocarcinoma metastasis from PC.

**Outcomes:**

The patient is currently alive without recurrence 44 months after resection for bone metastasis and 172 months after the initial surgery.

**Conclusion:**

Surgical resection may be favorable in patients with bone metastasis of PC that is well-controlled with chemotherapy.

## 1. Introduction

Pancreatic cancer (PC) is one of the most aggressive and metastatic malignant diseases, and the fourth leading cause of cancer-related deaths.^[1]^ Despite recent advancements in treatment, approximately 80% of patients develop metastases after curative resection.^[2]^ Several studies have demonstrated that liver metastasis and peritoneal dissemination account for most distant metastases, while bone metastasis is an uncommon recurrence after surgery for PC, accounting for approximately 3% of all recurrence sites.^[3,4]^

Conversion surgery is often performed in selected recurrent PC cases that are well-controlled with chemotherapy.^[5]^ Although the indicators for conversion surgery for recurrent PC remain unclear, good prognoses have been reported, mainly for lung metastases.^[6,7]^ However, there have been no reports of surgical intervention for bone metastases from PC, which is a rare site of recurrence.

We report a case of a patient who underwent multidisciplinary treatment, including resection of a solitary bone metastasis after curative surgery for PC, with long-term survival.

This study was approved by our Institutional Review Board and the requirement for written informed consent was waived (Approval Number: 20120443).

## 2. Case presentation

**Figure 1.**
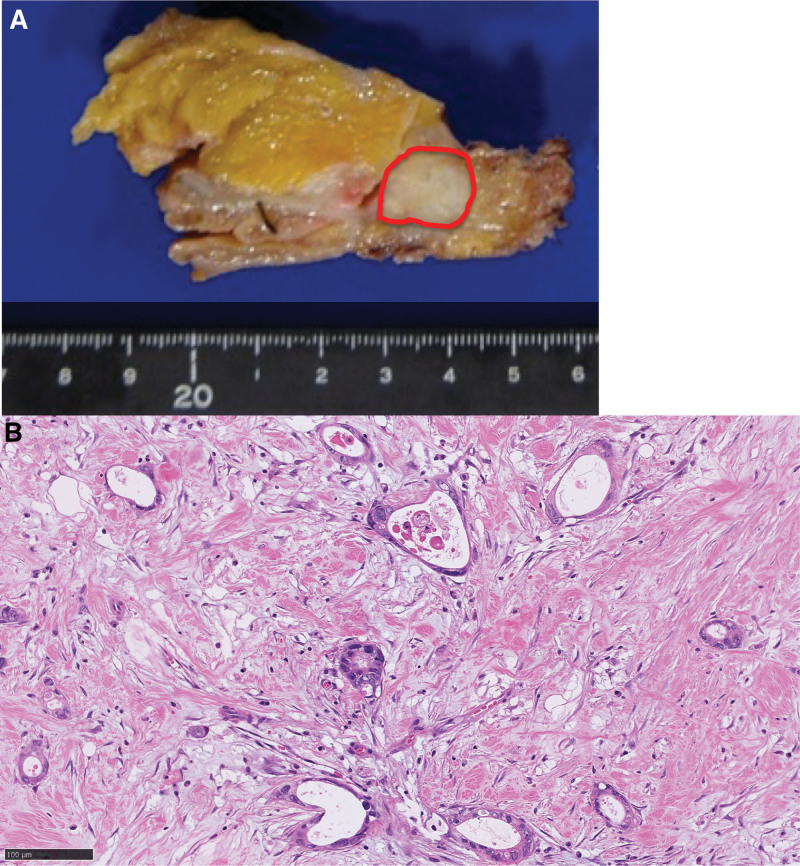
Pathological findings of pancreatic cancer. (A) The cut surface of the formalin-fixed specimen shows a 1.4 cm diameter whitish hard mass (indicated by the red circle). (B) Microscopic examination of hematoxylin–eosin-stained tissue shows moderately differentiated adenocarcinoma accompanied by abundant collagenous stroma.

The patient was a 60-year-old woman with pancreatic head cancer detected using computed tomography (CT), which was performed to investigate chest tightness. No evidence of distant metastasis was observed on CT. She underwent neoadjuvant chemoradiotherapy, comprising 4 cycles of chemotherapy—including continuous administration of 5-fluorouracil, cisplatin on days 5, 12, 19, and 26, mitomycin C on days 6, 13, 20, and 27, and heparin infusion—in addition to radiotherapy including a planned total dose of 40.0 Gy of external beam radiation therapy (40.0 Gy per 20 fractions).^[8,9]^ The patient’s carbohydrate antigen 19-9 (CA19-9) dropped from 93 to 46 U/mL. Subsequently, the patient underwent a pylorus-preserving pancreaticoduodenectomy. The pathological diagnosis was pancreatic ductal adenocarcinoma, moderately differentiated type, pT1c (20 × 13 × 12 mm), N0, M0, Ly1, V1, Pn1, pStage IA, according to the 8^th^ Union for International Cancer Control tumor–node–metastasis classification system, and histological assessment of therapeutic response was Tumor Regression Score 3 (Poor response) according to the College of American Pathologists grading system (Fig. 1). The patient received adjuvant therapy with 5-fluorouracil and heparin-based portal infusion chemotherapy combined with systemic administration of mitomycin C and CDDP for 4 weeks after surgery (PI4W).^[10]^

**Figure 2.**
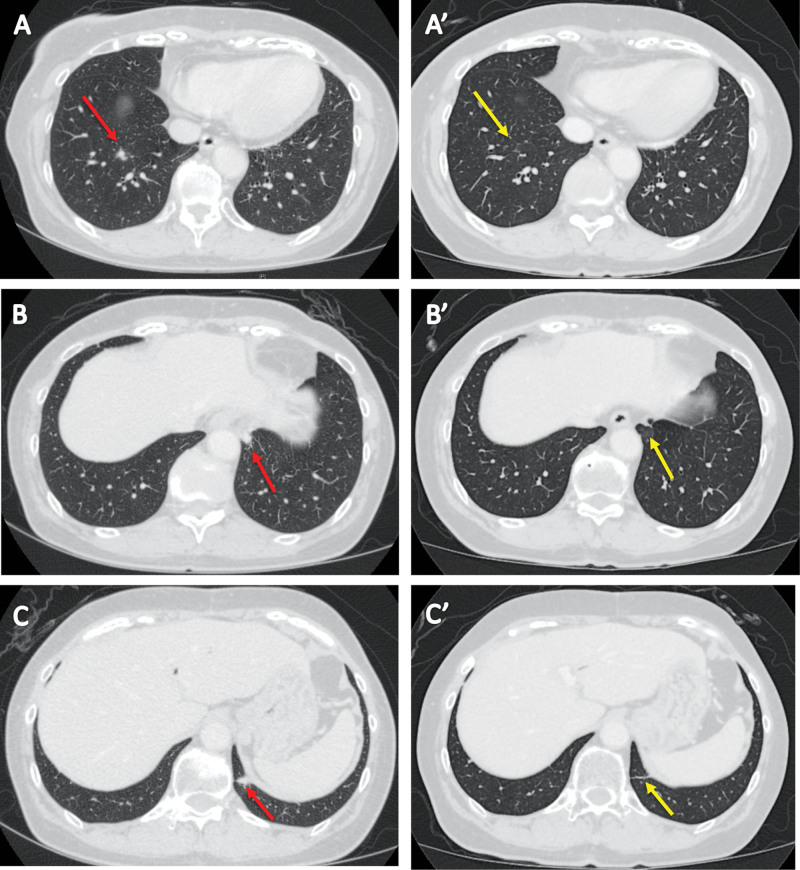
Computed tomography demonstrated multiple pulmonary nodules (red arrow), which were diagnosed as lung metastases of pancreatic cancer (A–C). These pulmonary nodules disappeared (yellow arrow) after chemotherapy (A’–C’).

At the 28-month follow-up, multiple pulmonary nodules were detected in both lungs via CT (Fig. 2A–C). The patient was diagnosed with recurrent lung metastases of PC, and gemcitabine was administered. Six months later, the chemotherapy regimen was changed from gemcitabine to S-1 because of adverse events. At 59 months after surgery, chemotherapy was terminated because all target lesions, which were multiple pulmonary nodules, had disappeared on imaging (Fig. 2A’–C’).

**Figure 3.**
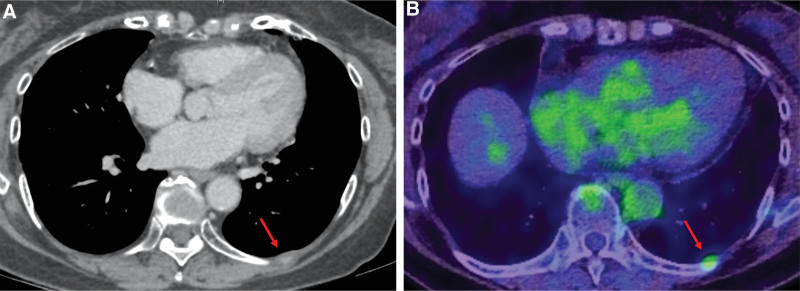
Computed tomography showed osteosclerotic changes (red arrow) in the left 9^th^ rib (A). This was diagnosed as recurrent solitary bone metastasis because positron emission tomography showed abnormal fluorine-18 fluorodeoxyglucose accumulation (red arrow) at the same site (B).

**Figure 4.**
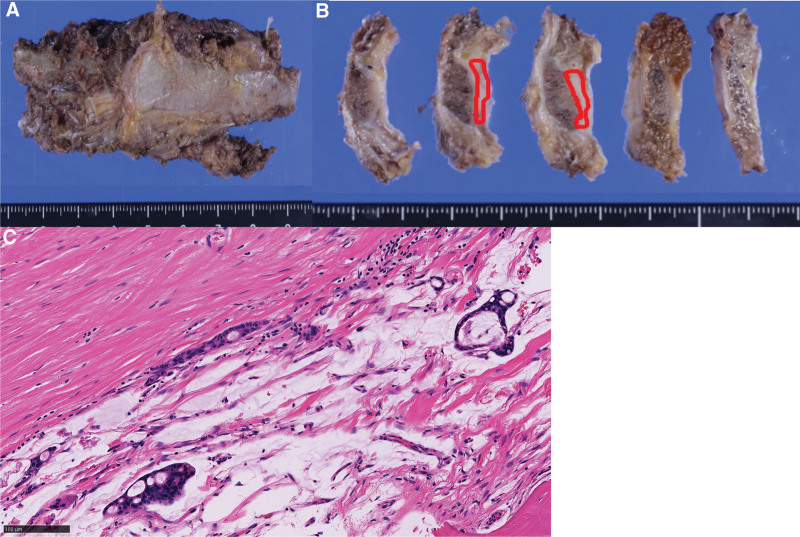
Pathological findings of solitary bone tumor. (A) Formalin-fixed surgical specimens of partial resection of the left 9^th^ rib. (B) The cut surface of formalin-fixed specimen: tumor location indicated by the red circle. (C) Hematoxylin–eosin-stained tissue of the bone mass shows atypical cells forming ill-defined glandular structures embedded in extracellular mucin. This finding is consistent with metastatic adenocarcinoma from pancreatic cancer.

At 77 months after surgery, positron emission tomography CT performed owing to elevated CA19-9 levels showed abnormal fluorine-18 fluorodeoxyglucose accumulation only in the left 9^th^ rib (Fig. 3). The patient was diagnosed with recurrent solitary bone metastasis and restarted on chemotherapy with S-1. Chemotherapy was continued intermittently until 128 months postoperatively, as the CA19-9 level declined after the resumption of S-1 but increased again when it was stopped. As the recurrence site was well-controlled for a long period and no other metastases were detected on CT or positron emission tomography CT, surgical resection of the solitary bone metastasis in the left 9^th^ rib was performed. Histopathological findings following thoracoscopic-assisted partial resection of the left 9^th^ rib revealed adenocarcinoma metastasis from the PC (Fig. 4). The patient was discharged 9 days after surgery without any complications. She continued to receive adjuvant chemotherapy with S-1, and there was no clinical evidence of recurrence 44 months after the resection for bone metastasis and 172 months after the initial surgery.

## 3. Discussion

We report a case of solitary bone metastasis following multiple lung metastases after radical resection of PC. Multidisciplinary treatment, including conversion surgery for solitary bone metastasis was performed, and long-term survival was achieved. To the best of our knowledge, this is the first report of long-term survival after surgical resection of bone metastases in PC.

Bone metastasis among PC patients is relatively rare compared to liver or peritoneal dissemination, and many biological behaviors of bone metastasis remain unclear.^[11,12]^ However, recent studies including population-based databases, such as the Surveillance, Epidemiology, and End Results database, have gradually clarified the characteristics of bone metastasis in PC.^[3,13–16]^ According to these studies, primary tumors of the pancreatic tail, compared to that of the pancreatic head, are more likely to develop bone metastases,^[13]^ and the spine is the most common site of bone metastasis in patients with PC.^[16]^ Based on the above results, in addition to the transarterial metastasis via the circulatory system, transvenous metastasis from the portal vein through the vertebral venous plexus may be involved in the mechanism of bone metastasis from PC.^[17]^ In this case, bone metastasis may have occurred through the portal vein because the primary tumor showed microvenous invasion.

The median overall survival for PC bone metastasis is poor, at approximately 6 months.^[15]^ However, in this case, the patient was able to undergo surgical resection and achieve long-term survival because the bone metastasis was well-controlled by long-term chemotherapy. Several reports have shown the effectiveness of conversion surgery for distant metastasis of PC, which was previously considered oncologically unresectable.^[18]^ In previous reports, the indicators for conversion surgery were long chemotherapy duration and low CA19-9 concentration.^[5,19]^ This case also met these criteria. Although there are no reports of surgical resection for bone metastasis of PC, because it is an uncommon metastatic site, surgical resection may be an option with strictly limited indications.

In previous reports, lung metastasis was an independent poor prognostic factor for bone metastasis of PC.^[13,14]^ A possible reason for this is that highly malignant bone metastasis had also spread to the lungs because some biomarkers, including IL-6, may be expressed at the same high levels in bone and lung metastases.^[17,20]^ However, PC lung metastasis has been reported to have a better prognosis than liver metastasis or peritoneal dissemination.^[4,21]^ When both lung and bone metastases are well-controlled with chemotherapy, as in this case, multidisciplinary treatment, including surgical resection, may provide a long-term prognosis. Further studies are required to explore new biomarkers that can be used to identify patients with distant metastases who respond to chemotherapy.^[22]^

## 4. Conclusions

We report a case of a patient who achieved long-term survival after surgical resection for a solitary bone metastasis following multiple lung metastases after radical resection of PC. Surgical resection may lead to favorable outcomes in patients with PC bone metastases that are well-controlled with chemotherapy.

## Acknowledgments

We would like to thank Editage (www.editage.com) for English language editing.

## Author contributions

**Conceptualization:** Minoru Kitago.

**Investigation:** Koki Hayashi.

**Methodology:** Minoru Kitago.

**Project administration:** Minoru Kitago.

**Supervision:** Minoru Kitago, Yuta Abe, Hiroshi Yagi, Yasushi Hasegawa, Shutaro Hori, Masayuki Tanaka, Yutaka Nakano, Keisuke Asakura, Yohei Masugi, Yuko Kitagawa.

**Visualization:** Koki Hayashi, Yohei Masugi.

**Writing—review & editing:** Minoru Kitago, Yuta Abe, Hiroshi Yagi, Yasushi Hasegawa, Shutaro Hori, Masayuki Tanaka, Yutaka Nakano, Keisuke Asakura, Yohei Masugi, Yuko Kitagawa.

**Writing—original draft:** Koki Hayashi.
